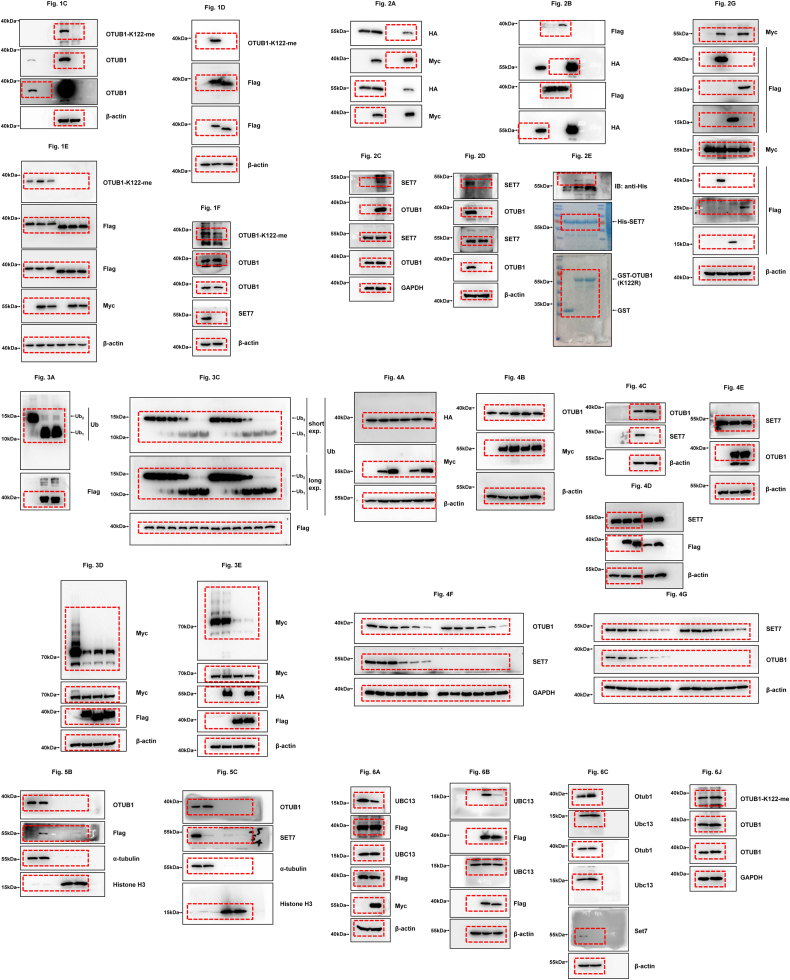# SET7 methylates the deubiquitinase OTUB1 at Lys ^122^ to impair its binding to E2 enzyme UBC13 and relieve its suppressive role on ferroptosis

**DOI:** 10.1016/j.jbc.2023.103054

**Published:** 2023-02-22

**Authors:** Hongyan Deng, Shuke Jia, Jinhua Tang, Fangjing Rong, Chenxi Xu, Xiaoyun Chen, Zixuan Wang, Chunchun Zhu, Xueyi Sun, Qian Liao, Wen Liu, Wenhua Li, Wuhan Xiao, Xing Liu

**Affiliations:** 1 College of Life Science, Wuhan University, Wuhan, P. R. China; 2 State Key Laboratory of Freshwater Ecology and Biotechnology, Institute of Hydrobiology, Chinese Academy of Sciences, Wuhan, P. R. China; 3 University of Chinese Academy of Sciences, Beijing, P. R. China; 4 Hubei Hongshan Laboratory, Wuhan, P. R. China; 5 The Innovation of Seed Design, Chinese Academy of Sciences, Wuhan, P. R. China

**Keywords:** SET7, OTUB1, methylation, UBC13, ferroptosis

## Abstract

The deubiquitinating enzyme OTUB1 possesses canonical deubiquitinase (DUB) activity and noncanonical, catalytic-independent activity, which has been identified as an essential regulator of diverse physiological processes. Posttranslational modifications of OTUB1 affect both its DUB activity and its noncanonical activity of binding to the E2 ubiquitin-conjugation enzyme UBC13, but further investigation is needed to characterize the full inventory of modifications to OTUB1. Here, we demonstrate that SET7, a lysine monomethylase, directly interacts with OTUB1 to catalyze OTUB1 methylation at lysine 122. This modification does not affect DUB activity of OTUB1 but impairs its noncanonical activity, binding to UBC13. Moreover, we found using cell viability analysis and intracellular reactive oxygen species assay that SET7-mediated methylation of OTUB1 relieves its suppressive role on ferroptosis. Notably, the methylation-mimic mutant of OTUB1 not only loses the ability to bind to UBC13 but also relieves its suppressive role on Tert-Butyl hydroperoxide–induced cell death and Cystine starvation/Erastin–induced cellular reactive oxygen species. Collectively, our data identify a novel modification of OTUB1 that is critical for inhibiting its noncanonical activity.

Ovarian tumor domain-containing ubiquitin aldehyde-binding protein 1 (OTUB1) is a member of OUT family of deubiquitylating enzymes (DUBs) (1). As the funding member of the OUT family, OTUB1 has been identified as an essential regulator of diverse physiological processes through deubiquitylating K48-linked ubiquitin chains (1, 2, 3, 4, 5, 6). To date, OTUB1 has been shown to catalyze deubiquitination of multiple targets including p100, ERα*,* UBE2E1*,* Snail*,* DEPTOR*,* YB-1*,* SMAD2/3*,* c-IAP1*,* p53*,* AKT*,* SOCS1*,* UBC13*,* PD-L1*,* Cyclin E1*,* MSH2*,* SLC7A11*,* TRAF3*,* TRAF6, and Nur77, resulting in positively or negatively modulating these targets' function (4, 7, 8, 9, 10, 11, 12, 13, 14, 15, 16, 17, 18, 19, 20, 21, 22, 23, 24, 25, 26). In addition to canonical DUB activity, the noncanonical, catalytic-independent activity of OTUB1 has been discovered (1, 27). OTUB1 inhibits the ubiquitination of target proteins by binding to and inhibiting the E2 ubiquitin-conjugation enzymes instead of directly removing K48-linked polyubiquitin chains on target proteins (4, 5, 6, 17). OTUB1 also inhibits DNA damage repair and promotes transforming growth factor-β signaling pathway independently of its DUB activity (12, 17). Recently, it is reported that the DUB activity is not required for OTUB1 to enhance SLC7A11 stabilization and inhibit ferroptosis (22).

The posttranslational modifications (PTMs) have been widely recognized for affecting target protein activity and function (28). Given that OTUB1 possesses deubiquitinase activity and noncanonical function activity, PTMs in affecting OTUB1 function at these two aspects have been identified (27). Factor inhibiting HIF regulates OTUB1 deubiquitinase activity by catalyzing OTUB1 hydroxylation in an oxygen-dependent manner (29, 30). The ubiquitin-like modifier FAT10 stimulates OTUB1 deubiquitinase activity in both covalent modification and a noncovalent manner (31). Meanwhile, PTMs in affecting the nonenzymatic activity OTUB1 have also been reported. UBCH5-mediated monoubiquitination of OTUB1 is required for its noncanonical regulation of p53 (32). Casein kinase 2 triggers OTUB1 nuclear localization by phosphorylating OTUB1 at Ser^16^ (33). S-Nitrosylation of OTUB1 alters its stability and UBC13 binding (34). Further investigating PTMs of OTUB1 will shed new lights on the molecular regulation of canonical and noncanonical activity of OTUB1.

SET7 (also known as SETD7, SET9, and SET7/9) is a monomethylase, which can methylate histone and nonhistone proteins, resulting in either negatively or positively modulating their target proteins' function (35, 36, 37, 38, 39, 40, 41, 42, 43, 44, 45). In this study, we found that that OTUB1 contains a SET7 targeting motif [(K/R) (S/T) K]. Further assays show that SET7 mediated the monomethylation of OTUB1 on lysine 122, leading to the impairment of its binding to UBC13 and the relief of its suppressive role on ferroptosis. This study reveals an essential role of SET7 in affecting the noncanonical activity of OTUB1.

## Results

### SET7 directly interacts with OTUB1 to methylate OTUB1 on lysine 122

**Figure 1:**
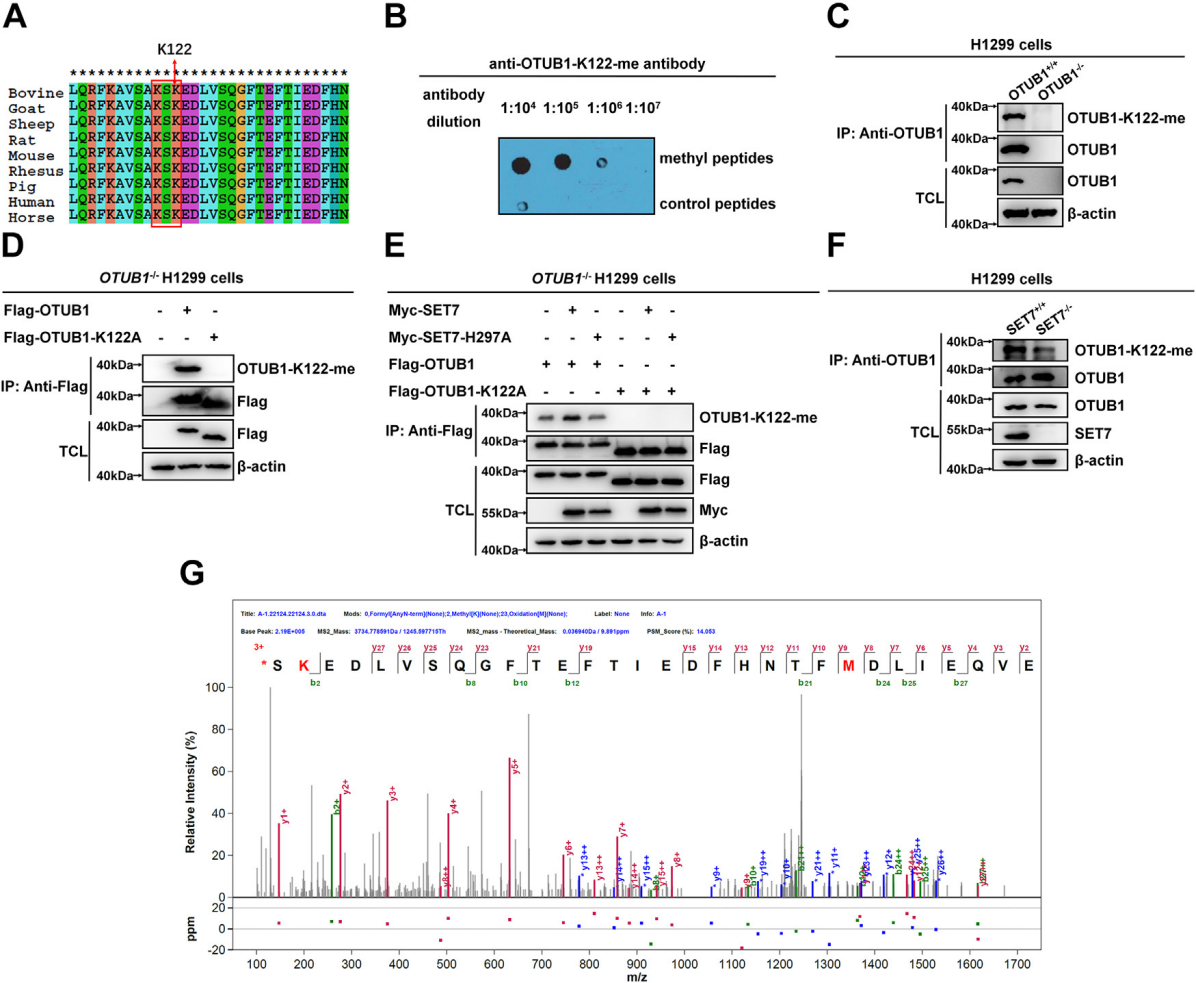
**SET7 methylates OTUB1 on lysine 122**. *A*, sequence alignment of partial OTUB1 proteins (111–140 amino acids) of human, mouse, rat, bovine, goat, sheep, rhesus, pig, and horse. The *red box* indicates a conserved consensus motif (R/K-S/T-K) methylated by SET7; the *red arrow* indicates the conserved lysine (K122) methylated by SET7. The amino acid is numbered based on human OTUB1 amino acid sequence. *B*, dot blot assay for the specificity of anti-OTUB1-K122-me antibody. Equal amounts of methylated-peptides or the control peptides were immunoblotted with the indicated dilution times of anti-OTUB1-K122-me antibody. *C*, Western blot analysis of endogenous methylated OTUB1 in *OTUB1*-deficient or WT H1299 cells (*OTUB1*^−/−^ or *OTUB1*^+/+^). Anti-OTUB1 antibody was used for immunoprecipitation and anti-OTUB1-K122-me antibody was used to detect monomethylated OTUB1. *D*, Western blot analysis of methylated OTUB1 in *OTUB1*^*−/−*^ H1299 cells transfected with Flag-OTUB1 or Flag-OTUB1-K122A. Anti-Flag antibody–conjugated agarose beads were used for immunoprecipitation and anti-OTUB1-K122-me antibody was used to detect monomethylated OTUB1. *E*, Western blot analysis of OTUB1 methylated by the WT SET7 or the enzymatic-deficient mutant of SET7 (H297A) in *OTUB1*^*−/−*^ H1299 cells transfected with indicated plasmids. Anti-Flag antibody–conjugated agarose beads were used for immunoprecipitation and anti-OTUB1-K122-me antibody was used to detect monomethylated OTUB1. *F*, Western blot analysis of endogenous methylated OTUB1 in *SET7*-deficient or WT H1299 cells (*SET7*^−/−^ or *SET7*^+/+^). Anti-OTUB1 antibody was used for immunoprecipitation. Anti-OTUB1-K122-me antibody was used to detect monomethylated OTUB1. *G*, the methylated residue in OTUB1 identified by mass spectrometry analysis. HEK293T cells were cotransfected with Flag-OTUB1 and Myc-SET7 plasmids. Cell lysate was immunoprecipitated with anti-Flag antibody–conjugated agarose beads overnight. Immunoprecipitated OTUB1 proteins were subjected to 8% SDS-PAGE gel, and OTUB1 bands were excised from the gel and analyzed by mass spectrometry.

In addition to histone H3, SET7 was also found to monomethylate nonhistone proteins by targeting a conserved core motif [(K/R) (S/T) K] in these proteins (37, 44, 46). After searching the amino acid sequences of OTUB1 from different organisms, the ' KSK ' residues were identified in OTUB1 (Fig. 1*A*), in which lysine 122 might be methylated by SET7. To validate this hypothesis, we developed an antibody (anti-OTUB1-K122me) to specifically recognize lysine 122 monomethylation of human OTUB1. The specificity of this antibody was validated by dot blot assay (Fig. 1*B*). Monomethylation of OTUB1 was readily detected by the antibody in WT H1299 cells (*OTUB1*^+/+^) but not in *OTUB1*-deficient H1299 cells (*OTUB1*^−/−^) (Fig. 1*C*). However, reconstitution of Flag-tagged WT *OTUB1* in *OTUB1*^−/−^ H1299 cells restored the methylated OTUB1 to be detected (Fig. 1*D*). By contrast, reconstitution of methylation-site mutant of OTUB1 (OTUB1-K122A) could not restore the methylated OTUB1 (Fig. 1*D*).

Subsequently, we examined whether OTUB1 methylation at K122 was mediated by SET7. When Flag-tagged WT OTUB1 (Flag-OTUB1) was cotransfected with empty vector, Myc-SET7 or its enzymatically deficient mutant (Myc-SET7-H297A) (37) in *OTUB1*^−/−^ H1299 cells, monomethylation of OTUB1 in the cells transfected with WT SET7 was higher than in the cells transfected with empty vector control, while monomethylation of OTUB1 in the cells transfected with Myc-SET7-H297A was similar to the cells transfected with empty vector control, suggesting that OTUB1 might be methylated by endogenous SET7 in these cells or OUTB1 might be methylated at K122 by another unknown methyltransferase (Fig. 1*E*). However, the methylated OTUB1 was not detected when Flag-tagged OTUB1-K122A (Flag-OTUB1-K122A) was cotransfected with SET7 or its enzymatically deficient mutant (SET7-H297A) in *OTUB1*^−/−^ H1299 cells (Fig. 1*E*). To further determine whether endogenous OTUB1 was methylated by SET7, we examined methylation of OTUB1 in *SET7*^+/+^ and *SET7*^−/−^ H1299 cells after coimmunoprecipitation with anti-OTUB1 antibody. Monomethylation of OTUB1 in *SET7*^+/+^ H1299 cells was higher than that in *SET7*^−/−^ H1299 cells (Fig. 1*F*). To further validate these results, we performed mass spectrometry assay. As shown in Figure 1*G*, the monomethylated K122 was identified in OTUB1 when SET7 was coexpressed (Fig. 1*G*).

**Figure 2:**
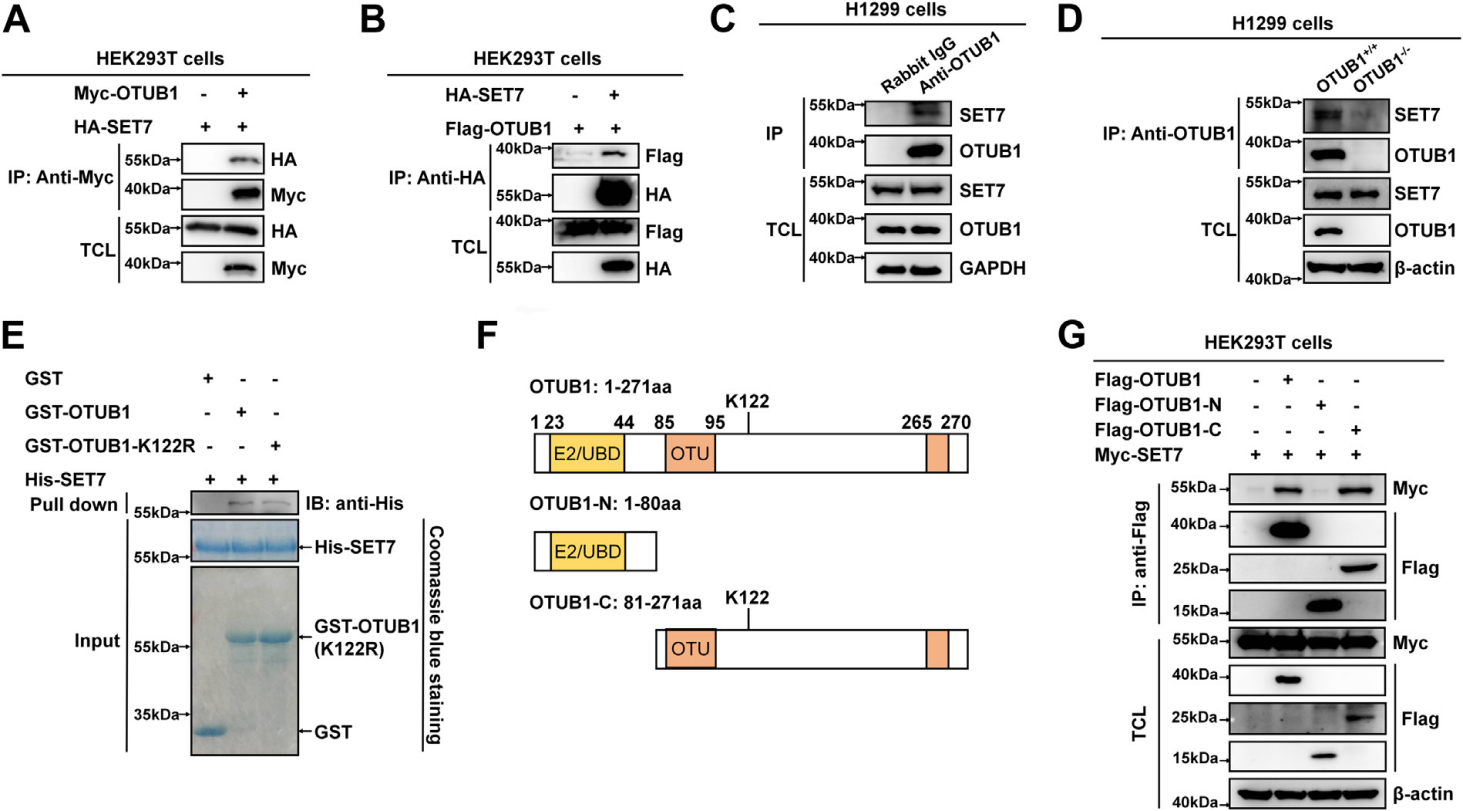
**SET7 interacts with OTUB1**. *A*, coimmunoprecipitation analysis of Myc-OTUB1 with HA-SET7. HEK293T cells were cotransfected with indicated plasmids for 24 h. Anti-Myc antibody–conjugated agarose beads were used for immunoprecipitation, and the interaction was detected by immunoblotting with the indicated antibodies. *B*, coimmunoprecipitation analysis of HA-SET7 with Flag-OTUB1. HEK293T cells were cotransfected with indicated plasmids for 24 h. Anti-HA antibody–conjugated agarose beads were used for immunoprecipitation, and the interaction was detected by immunoblotting with the indicated antibodies. *C*, endogenous interaction between OTUB1 and SET7 in H1299 cells. Anti-OTUB1 antibody was used for immunoprecipitation, and normal rabbit IgG was used as a control. *D*, endogenous interaction between OTUB1 and SET7 in the WT (*OTUB1*^+/+^) or *OTUB1*-deficient (*OTUB1*^−/−^) H1299 cells. Anti-OTUB1 antibody was used for immunoprecipitation, and the interaction was detected by immunoblotting with anti-SET7 antibody. *E*, GST pull-down assay for GST-tagged OTUB1 or GST-tagged OTUB1-K122R and His-tagged SET7. GST-tagged OTUB1, GST-tagged OTUB1-K122R, and His-tagged SET7 were expressed in *Escherichia coli* (BL21), respectively. The association of GST-OTUB1 or GST-OTUB1-K122R with His-SET7 was detected by immunoblotting with anti-His antibody. His-SET7, GST, GST-OTUB1, and GST-OTUB1-K122R proteins were stained with Coomassie Blue. *F*, schematic of OTUB1 domains and OTUB1 domain mutants. *G*, coimmunoprecipitation analysis of Myc-SET7 with Flag-OTUB1–truncated mutants. HEK293T cells were cotransfected with the indicated plasmids. Anti-Flag antibody–conjugated agarose beads were used for immunoprecipitation, and the interaction was analyzed by immunoblotting with the indicated antibodies. Flag-OTUB1 fragments (OTUB1-N, 1–80 aa; OTUB1-C, 81–271 aa).

Next, we examined whether OTUB1 interacts with SET7 by coimmunoprecipitation assay. Ectopically expressed OTUB1 interacted with ectopically expressed SET7 and vice versa (Fig. 2, *A* and *B*). In H1299 cells, endogenous OTUB1 was coimmunoprecipitated with endogenous SET7 (Fig. 2*C*), while endogenous coimmunoprecipitation between OTUB1 and SET7 was not detected in *OTUB1*-deficient H1299 cells (Fig. 2*D*). *Escherichia coli*–expressed GST-tagged OTUB1 or GST-OTUB1-K122R interacted with *E. coli*–expressed His-tagged SET7 *in vitro* (Fig. 2*E*). These data indicated that OTUB1 directly associated with SET7. In addition, it appeared that the N-terminal domain (E2/UBD) of OTUB1 did not bind to SET7, while the C-terminal domain of OTUB1 could bind to SET7 (Fig. 2, *F* and *G*).

Taken together, these data suggest that SET7 interacts with OTUB1 to catalyze monomethylation of OTUB1 at K122.

### SET7-mediated methylation of OTUB1 does not affect enzymatic activity, protein stability, and subcellular location of OTUB1

**Figure 3:**
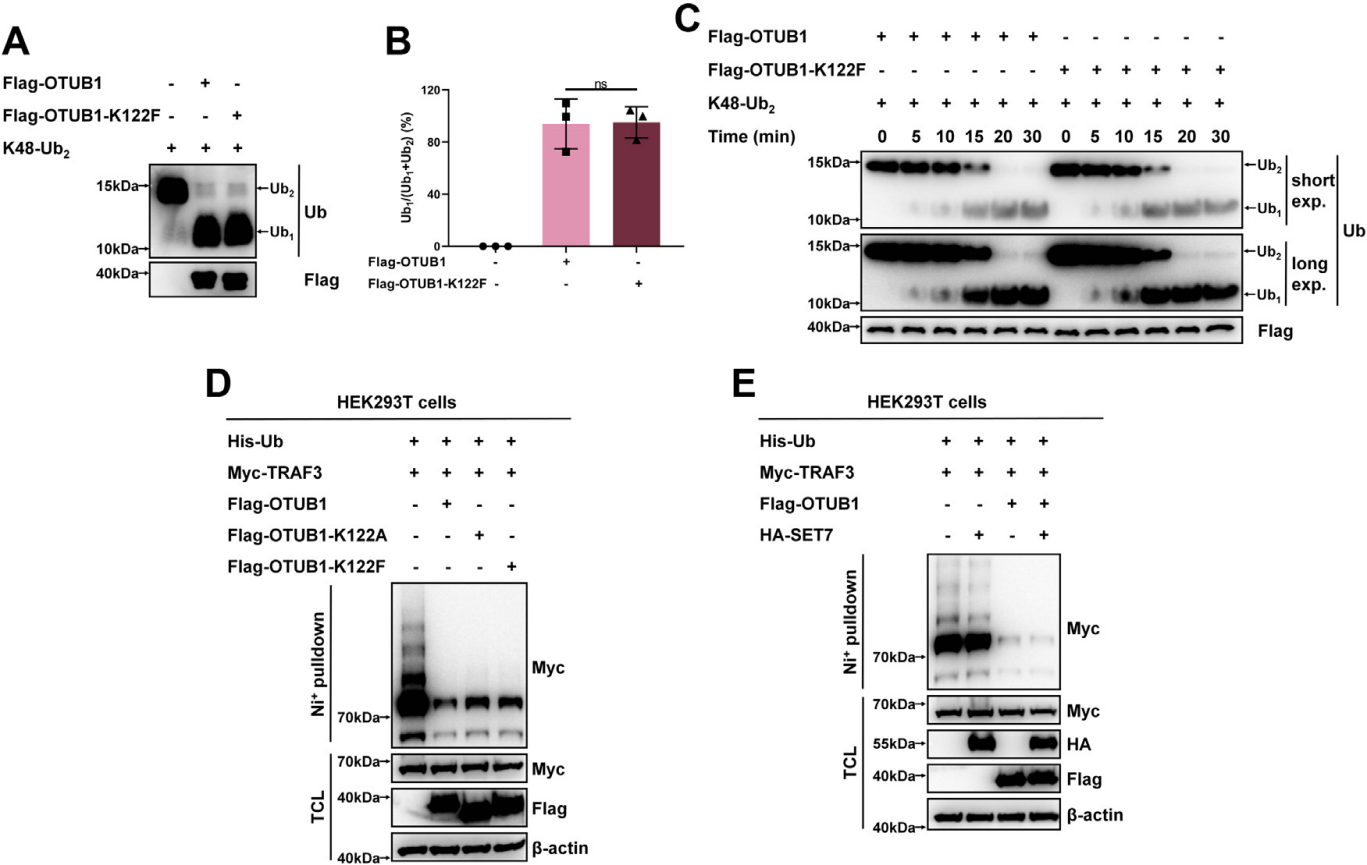
**SET7-mediated methylation of OTUB1 does not affect deubiquitinase activity of OTUB1**. *A*, analysis of catalytic activity of Flag-OTUB1 or its methylation-mimic mutant (Flag-OTUB1-K122F) on K48 di-Ub chain. *B*, the enzymatic activities in (*A*) were determined by normalizing the intensities of ubiquitin to the intensities of ubiquitin plus di-ubiquitin. Data show mean ± SD; Student’s two-tailed *t* test; Data from three independent experiments. *C*, a time course analysis of the catalytic activity of Flag-OTUB1 or its methylation-mimic mutant (Flag-OTUB1-K122F) on K48 di-Ub chain. *D*, TRAF3 ubiquitination in HEK293T cells transfected with Myc-TRAF3, His-Ub, together with Flag-OTUB1, Flag-OTUB1-K122A, or Flag-OTUB1-K122F (Flag empty vector was used as a control) for 24 h. *E*, TRAF3 ubiquitination in HEK293T cells transfected with Myc-TRAF3, His-Ub, Flag-OTUB1, or Flag empty vector used as a control, together with HA-SET7 or HA empty vector used as a control for 24 h.

Given that OTUB1 is a typical deubiquitinase, we initially sought to know whether SET7-mediated methylation of OTUB1 at K122 could affect deubiquitinase activity of OTUB1. We compared the deubiquitinase activity between WT OTUB1 and its methylation-mimic mutant (OTUB1-K122F) by enzymatic activity assay. The WT OTUB1 had the same capability as its methylation-mimic mutant (OTUB1-K122F) to catalyze deubiquitination of K48-linked di-ubiquitin (Fig. 3, *A*–*C*). Then, we examined the effect of OTUB1 on ubiquitination of TRAF3, a well-defined target deubiquitinated by OTUB1 (47). As shown in Figure 3*D*, the deubiquitination activity of WT OTUB1 on TRAF3 was similar to that of the two mutants, OTUB1-K122A and OTUB1-K122F. In addition, coexpression of SET7 had no obvious effect on the deubiquitination of TRAF3 by OTUB1 (Fig. 3*E*). These data suggest that SET7-mediated methylation of OTUB1 does not affect deubiquitinase activity of OTUB1.

**Figure 4:**
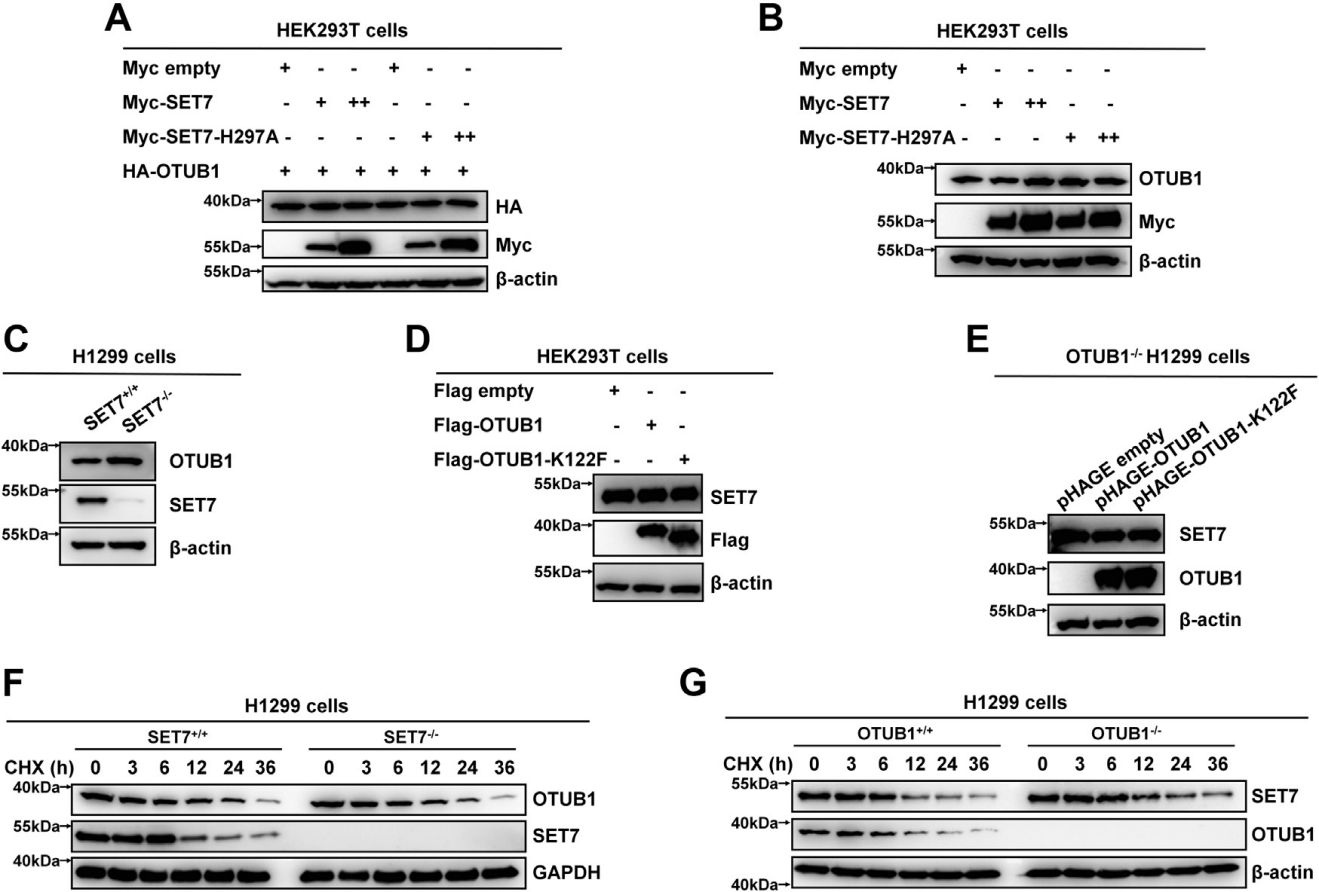
**SET7-mediated methylation of OTUB1 does not affect OTUB1 protein stability and *vice versa***. *A*, Western blot analysis of HA-OTUB1 expression in HEK293T cells cotransfected with an increasing amount of Myc-SET7 or the enzymatically deficient mutant (Myc-SET7-H297A) expression plasmid. *B*, Western blot analysis of endogenous OTUB1 expression in HEK293T cells transfected with an increasing amount of Myc-SET7 or the enzymatically deficient mutant (Myc-SET7-H297A) expression plasmid. *C*, Western blot analysis of endogenous OTUB1 expression in *SET7*-deficient or WT H1299 cells (*SET7*^−/−^ or *SET7*^+/+^). *D*, Western blot analysis of endogenous SET7 expression in HEK293T cells transfected with Flag-OTUB1 or the methylation-mimic mutant (Flag-OTUB1-K122F) expression plasmid. *E*, Western blot analysis of endogenous SET7 expression in *OTUB1*-deficient H1299 cells (*OTUB1*^−/−^) reconstituted with *OTUB1* or its methylation-mimic mutant (OTUB1-K122F) by lentivirus. *F*, Western blot analysis of endogenous OTUB1 expression in *SET7*-deficient or WT H1299 cells (*SET7*^−/−^ or *SET7*^+/+^) treated with CHX (50 μg/ml) for the indicated times. *G*, Western blot analysis of endogenous SET7 expression in *OTUB1*-deficient or WT H1299 cells (*OTUB1*^−/−^ or *OTUB1*^+/+^) treated with CHX (50 μg/ml) for the indicated times. CHX, cycloheximide.

Subsequently, we sought to know whether SET7-mediated methylation of OTUB1 at K122 could affect OTUB1 protein stability. Overexpression of either SET7 or its enzymatically deficient mutant (SET7-H297A) with increasing amounts together with HA-tagged OTUB1 in HEK293T cells had no obvious effect on OTUB1 protein stability (Fig. 4*A*). Endogenous OTUB1 protein level was not changed when Myc-SET7 or its enzymatically deficient mutant (SET7-H297A) were transfected into HEK293T cells with increasing amounts (Fig. 4*B*). Moreover, endogenous OTUB1 protein level was almost the same between *SET7*^+/+^ and *SET7*^−/−^ H1299 cells (Fig. 4*C*). Moreover, overexpression of WT OTUB1 and its methylation-mimic mutant (OTUB1-K122F) also did not alter endogenous SET7 protein level in HEK293T cells (Fig. 4*D*). In *OTUB1*^−/−^ H1299 cells, reconstitution of WT *OTUB1* or its methylation-mimic mutant (OTUB1-K122F) had no effect on endogenous SET7 protein levels (Fig. 4*E*). Consistently, by cycloheximide pulse chase assay, *SET7* deficiency had no effect on endogenous OTUB1 protein levels (Fig. 4*F*), meanwhile, *OTUB1* deficiency also did not affect endogenous SET7 protein levels (Fig. 4*G*). These data suggest that SET7 and OTUB1 do not affect each other at protein level although they directly associate in cells.

**Figure 5:**
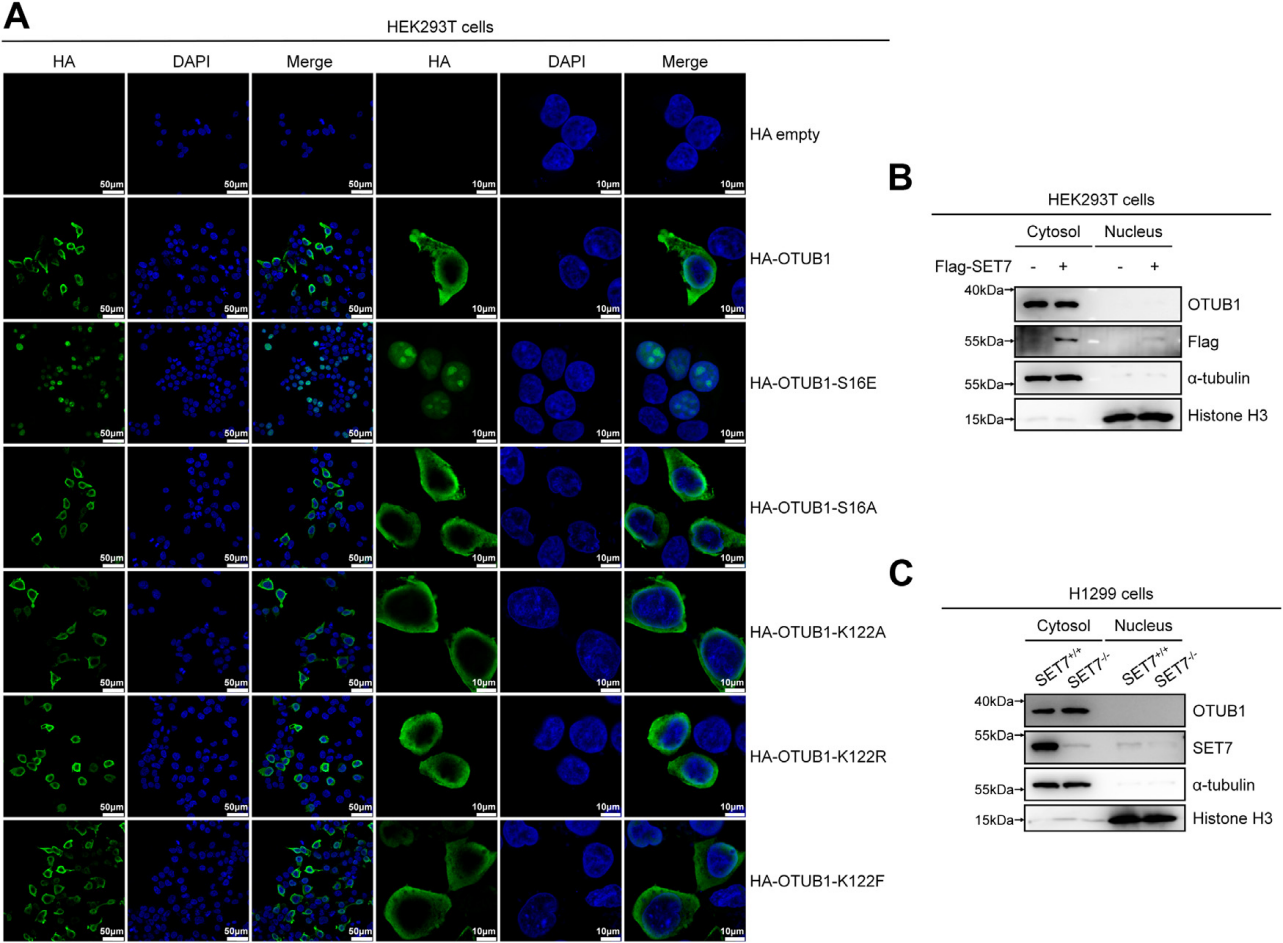
**SET7-mediated methylation of OTUB1 does not affect OTUB1 cellular localization**. *A*, HEK293T cells were transfected with HA empty, HA-OTUB1, HA-OTUB1-S16E, HA-OTUB1-S16A, HA-OTUB1-K122A, HA-OTUB1-K122R, or HA-OTUB1-K122F expression plasmid. Confocal microscopy image of exogenous OTUB1 was detected by immunofluorescence staining using anti-HA antibody. Scale bar represents 50 μm (in the first three columns) or 10 μm (in the last three columns) as indicated. *B*, nucleic-cytoplasmic separation analysis of cellular localization of endogenous OTUB1 in HEK293T cells transfected with or without Flag-SET7 for 24 h. *C*, nucleic-cytoplasmic separation analysis of cellular localization of endogenous OTUB1 in *SET7*-deficient or WT H1299 cells (*SET7*^−/−^ or *SET7*^+/+^).

As reported previously, phosphorylation of OTUB1 at Serine 16 by Casein kinase 2 causes nuclear accumulation of OTUB1 (33), we sought to know whether SET7-mediated methylation of OTUB1 at K122 could alter subcellular localization of OTUB1. By immunofluorescent staining, we detected that WT HA-OTUB1 mainly located in cytosol when overexpressed in HEK293T cells (Fig. 5*A*). In agreement with the previous report (33), the phosphomimic mutant of OTUB1, HA-OTUB1-S16E, localized almost exclusively to nuclei when overexpressed in HEK293T cells, whereas the nonphosphorylatable mutant of OTUB1, HA-OTUB1-S16A located entirely in cytosol when overexpressed in HEK293T cells (Fig. 5*A*). However, the methylation-deficient mutants of OTUB1, HA-OTUB1-K122A or HA-OTUB1-K122R, mainly located in cytosol, exactly the same as the methylation-mimic mutant of OTUB1, HA-OTUB1-K122F (Fig. 5*A*). Moreover, OTUB1 was detected primarily in the cytoplasmic fraction, while overexpression of SET7 did not cause its nuclear translocation (Fig. 5*B*). In fact, the subcellular localization of OTUB1 was almost the same between *SET7*^+/+^ and *SET7*^−/−^ H1299 cells (Fig. 5*C*). These data suggest that SET7-mediated methylation of OTUB1 does not affect the subcellular localization of OTUB1.

### SET7-mediated methylation of OTUB1 attenuates OTUB1 binding to UBC13

**Figure 6:**
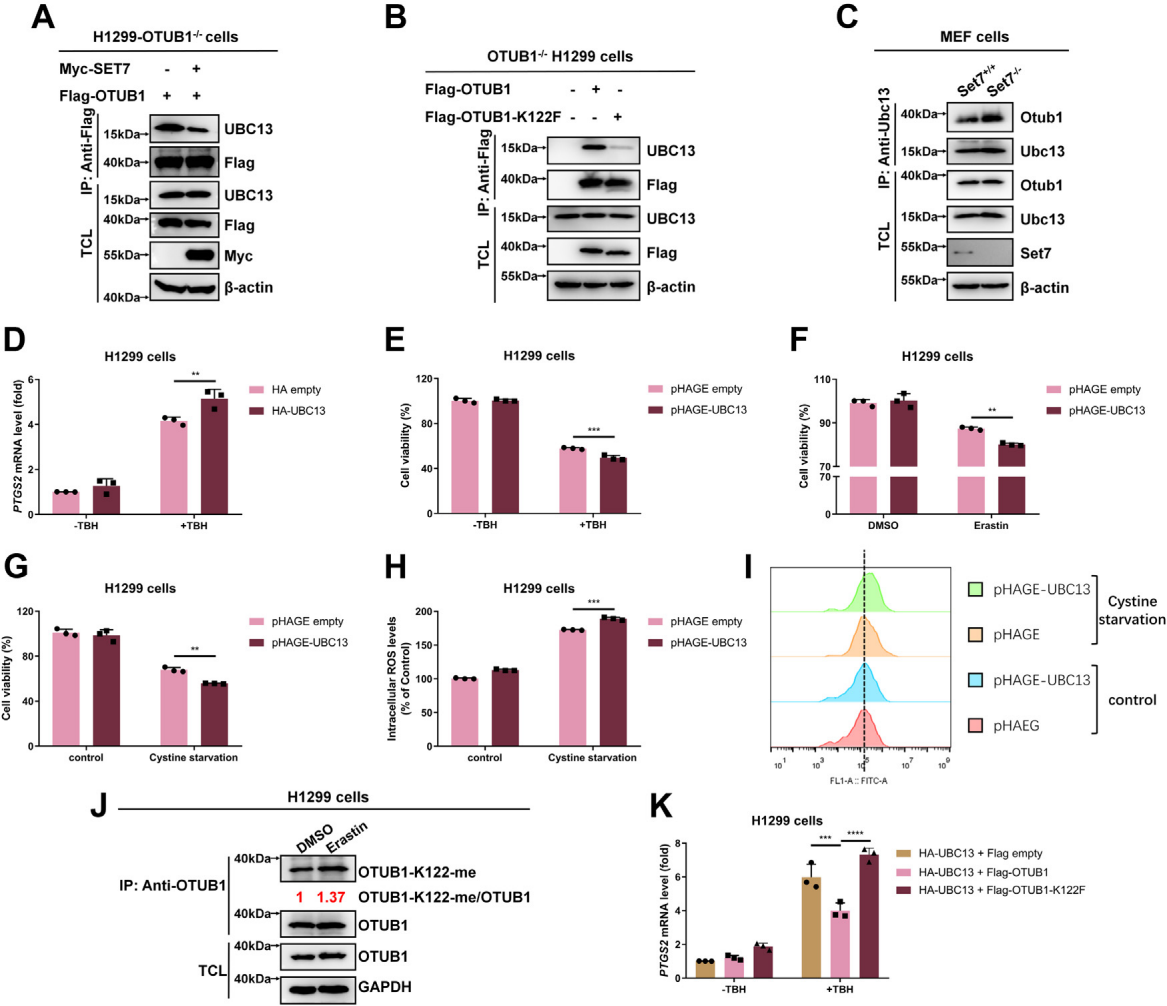
**SET7-mediated methylation of OTUB1 suppresses OTUB1 binding to UBC13**. *A*, coimmunoprecipitation of Flag-OTUB1 with endogenous UBC13 when SET7 were overexpressed. *OTUB1*-deficient H1299 cells (*OTUB1*^−/−^) were transfected with indicated plasmids for 24 h. Anti-Flag antibody–conjugated agarose beads were used for immunoprecipitation, and the interaction was detected by immunoblotting with the indicated antibodies. *B*, coimmunoprecipitation of Flag-OTUB1 or the methylation-mimic mutant (Flag-OTUB1-K122F) with endogenous UBC13. *OTUB1*-deficient H1299 cells (*OTUB1*^−/−^) were transfected with indicated plasmids for 24 h. Anti-Flag antibody–conjugated agarose beads were used for immunoprecipitation, and the interaction was detected by immunoblotting with the indicated antibodies. *C*, endogenous interaction between Otub1 and Ubc13 in *Set7*-deficient or WT MEF cells (*Set7*^−/−^ or *Set7*^+/+^). Anti-Ubc13 antibody was used for immunoprecipitation. *D*, qPCR analysis of *PTGS2* mRNA in H1299 cells transfected with HA-UBC13 or HA empty as a control and treated with or without TBH (100 μM) for 7 h. Two-way ANOVA analysis; Data show mean ± SD; Tukey’s multiple comparisons test; ∗∗Adjusted *p* < 0.01; Data from three independent experiments. *E*, cell viability of H1299 cells with or without UBC13 overexpression infected by lentivirus and treated with or without TBH (200 μM) for 7 h and examined by CCK8 assay. Two-way ANOVA analysis; Data show mean ± SD; Tukey’s multiple comparisons test; ∗∗∗Adjusted *p* < 0.001; Data from three independent experiments. *F*, cell viability of H1299 cells with or without UBC13 overexpression infected by lentivirus and treated with DMSO (as a control) or Erastin (20 μM) for 24 h and examined by CCK8 assay. Two-way ANOVA analysis; Data show mean ± SD; Tukey’s multiple comparisons test; ∗∗Adjusted *p* < 0.01; Data from three independent experiments. *G*, cell viability of H1299 cells with or without UBC13 overexpression infected by lentivirus and treated with or without cystine starvation for 24 h and examined by CCK8 assay. Two-way ANOVA analysis; Data show mean ± SD; Tukey’s multiple comparisons test; ∗∗Adjusted *p* < 0.01; Data from three independent experiments. *H* and *I*, intracellular ROS in H1299 cells with or without UBC13 overexpression infected by lentivirus and treated with or without Cystine starvation for 24 h and examined by flow cytometry. Quantification of the intracellular ROS levels in (*H*) and representative flow cytometry histogram in (*I*). Two-way ANOVA analysis; Data show mean ± SD; Tukey’s multiple comparisons test; ∗∗∗Adjusted *p* < 0.001; Data from three independent experiments. *J*, Western blot analysis of endogenous methylated OTUB1 in H1299 cells treated with Erastin (20 μM) or DMSO as a control. Anti-OTUB1 antibody was used for immunoprecipitation and anti-OTUB1-K122-me antibody was used to detect monomethylated OTUB1. *K*, qPCR analysis of *PTGS2* mRNA in H1299 cells transfected with HA-UBC13 together with Flag-OTUB1, Flag-OTUB1-K122F, or Flag empty as a control and treated with or without TBH (100 μM) for 7 h. Two-way ANOVA analysis; Data show mean ± SD; Tukey’s multiple comparisons test; ∗∗∗Adjusted *p* < 0.001, ∗∗∗∗Adjusted *p* < 0.0001; Data from three independent experiments. CCK8, Cell Counting Kit-8; MEF, mouse embryo fibroblast; qPCR, quantitative real-time PCR; ROS, reactive oxygen species; TBH, Tert-Butyl hydroperoxide.

It is well-defined that OTUB1 also possesses a noncanonical function independently of its deubiquitinase activity, which inhibits the ubiquitination of target proteins by binding to and inhibiting the E2 ubiquitin-conjugation enzymes (4, 5, 6). Thus, we sought to know whether SET7-mediated methylation of OTUB1 can affect OTUB1 binding to the E2 enzyme, UBC13. Ectopically expressed SET7 inhibited the interaction between reconstituted OTUB1 with endogenous UBC13 in *OTUB1*^−/−^ H1299 cells (Fig. 6*A*). Furthermore, in *OTUB1*^−/−^ H1299 cells, reconstitution of WT OTUB1 bound to more endogenous UBC13 than reconstitution of the methylation-mimic mutant of OTUB1 (K122F) (Fig. 6*B*). In addition, coimmunoprecipitation assay showed that endogenous Ubc13 bound to more endogenous Otub1 in *Set7*-deficient mouse embryo fibroblast (MEF) cells (*Set7*^−/−^) than in *Set7*-intact MEF cells (*Set7*^+/+^) (Fig. 6*C*).

These data suggest that SET7-mediated methylation of OTUB1 attenuates OTUB1 binding to UBC13.

### SET7-mediated methylation of OTUB1 relieves the suppressive role of OTUB1 on ferroptosis

Recently, OTUB1 was identified as a negative regulator of ferroptosis by directly interacting with and stabilizing SLC7A11, which was independent of its DUB activity (22). Since OTUB1 possesses a noncanonical function independently of its DUB activity by binding to and inhibiting the E2 ubiquitin-conjugation enzymes, such as UBC13 (4, 5, 6), SET7-mediated methylation of OTUB1 attenuates OTUB1 binding to UBC13. Therefore, we sought to determine whether SET7-mediated methylation of OTUB1 has an impact on ferroptosis.

Firstly, we investigated the role of UBC13 in ferroptosis. We checked the expression of *PTGS2*, a marker of ferroptosis (22). In response to Tert-Butyl hydroperoxide (TBH) treatment (33), overexpression of UBC13 promoted the induction of *PTGS2* mRNA dramatically (Fig. 6*D*). Then, we examined the role of UBC13 in reactive oxygen species (ROS)-induced ferroptosis. As shown in Figure 6*E*, treatment with TBH caused dramatic cell death, and overexpression of UBC13 induced cell death upon TBH treatment. Similar results were obtained when the cells were treated with Erastin, a small molecule that induces ferroptosis by blocking the transmembrane cystine/glutamate antiporter system xCT (22) (Fig. 6*F*) or treated by Cystine starvation (Fig. 6*G*). In agreement with these observations, UBC13 overexpression upregulated the ROS levels induced by Cysteine starvation (Fig. 6, *H* and *I*). In addition, methylation of OTUB1 was induced by Erastin treatment (Fig. 6*J*). Co-overexpression of WT OTUB1 with UBC13 in H1299 cells suppressed the induction of *PTGS2* mRNA significantly, but co-overexpression of the methylation-mimic mutant of OTUB1 (K122F) relieved this suppressive effect (Fig. 6*K*).

**Figure 7:**
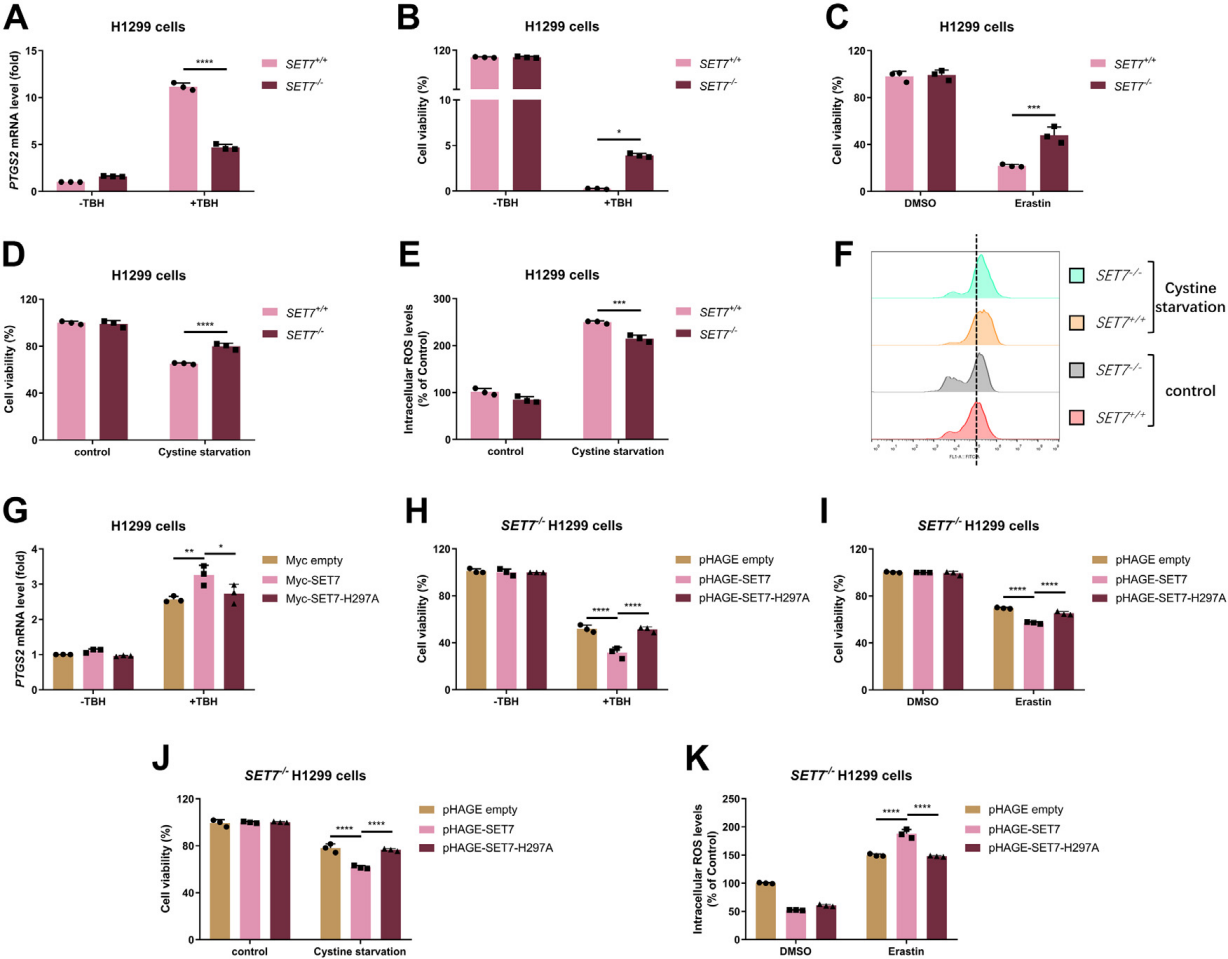
**SET7 promotes cellular ferroptosis dependent of its methyltransferase activity**. *A*, qPCR analysis of *PTGS2* mRNA in *SET7*-deficient or WT H1299 cells (*SET7*^−/−^ or *SET7*^+/+^) and treated with or without TBH (100 μM) for 7 h. *B*, cell viability of *SET7*-deficient or WT H1299 cells (*SET7*^−/−^ or *SET7*^+/+^) and treated with or without TBH (200 μM) for 7 h and examined by CCK8 assay. *C*, cell viability of *SET7*-deficient or WT H1299 cells (*SET7*^−/−^ or *SET7*^+/+^) and treated with DMSO (as a control) or Erastin (20 μM) for 24 h and examined by CCK8 assay. *D*, cell viability of *SET7*-deficient or WT H1299 cells (*SET7*^−/−^ or *SET7*^+/+^) and treated with or without cystine starvation for 24 h and examined by CCK8 assay. *E* and *F*, intracellular ROS in *SET7*-deficient or WT H1299 cells (*SET7*^−/−^ or *SET7*^+/+^) and treated with or without Cystine starvation for 24 h and examined by flow cytometry. Quantification of the intracellular ROS levels in (*E*) and representative flow cytometry histogram in (*F*). *G*, qPCR analysis of *PTGS2* mRNA in H1299 cells transfected with indicated plasmids and treated with or without TBH (100 μM) for 7 h. *H*, cell viability of *SET7*-deficient H1299 cells (*SET7*^*−/−*^) (n = 3) reconstituted with *SET7* or its enzymatically deficient mutant (SET7-H297A) by lentivirus and treated with or without TBH (200 μM) for 7 h and examined by CCK8 assay. *I*, cell viability of *SET7*-deficient H1299 cells (*SET7*^*−/−*^) (n = 3) reconstituted with *SET7* or its enzymatically deficient mutant (SET7-H297A) by lentivirus and treated with DMSO (as a control) or Erastin (20 μM) for 24 h and examined by CCK8 assay. *J*, cell viability of *SET7*-deficient H1299 cells (*SET7*^*−/−*^) (n = 3) reconstituted with *SET7* or its enzymatically deficient mutant (SET7-H297A) by lentivirus and treated with or without cystine starvation for 24 h and examined by CCK8 assay. *K*, intracellular ROS in *SET7*-deficient H1299 cells (*SET7*^*−/−*^) (n = 3) reconstituted with *SET7* or its enzymatically deficient mutant (SET7-H297A) by lentivirus and treated with DMSO (as a control) or Erastin (20 μM) for 24 h and examined by flow cytometry. Two-way ANOVA analysis; Data show mean ± SD; Tukey’s multiple comparisons test; ∗Adjusted *p* < 0.05, ∗∗Adjusted *p* < 0.01, ∗∗∗Adjusted *p* < 0.001, ∗∗∗∗Adjusted *p* < 0.0001; Data from three independent experiments. CCK8, Cell Counting Kit-8; qPCR, quantitative real-time PCR; ROS, reactive oxygen species; TBH, Tert-Butyl hydroperoxide.

Subsequently, we examined the role of SET7 in ferroptosis. In response to TBH, *SET7* deficiency in H1299 cells largely suppressed the induction of *PTGS2* mRNA (Fig. 7*A*). *SET7* deficiency in H1299 cells relieved cells from TBH-induced ferroptosis significantly (Fig. 7*B*). Similar results were obtained when the cells were treated with Erastin (Fig. 7*C*) or treated by Cystine starvation (Fig. 7*D*). In agreement with these observations, the ROS levels induced by Cysteine starvation were largely reduced in *SET7*^−/−^ H1299 cells than that in WT H1299 cells (*SET7*^+/+^) (Fig. 7, *E* and *F*). Furthermore, in response to TBH, overexpression of WT SET7 in H1299 cells promoted the induction of *PTGS2* mRNA, while overexpression of the enzymatically deficient mutant of SET7 did not (Fig. 7*G*). Reconstitution of WT SET7 significantly promoted cell ferroptosis induced by TBH stimulation, Erastin treatment, or Cystine starvation, but reconstitution of the enzymatically deficient mutant of SET7 (H297A) lost this effect (Fig. 7, *H*–*J*). In addition, the ROS levels induced by Erastin treatment were upregulated significantly in *SET7*^−/−^ H1299 cells reconstituted with the WT SET7 but not in *SET7*^−/−^ H1299 cells reconstituted with the enzymatically deficient mutant of SET7 (H297A) (Fig. 7*K*).

**Figure 8:**
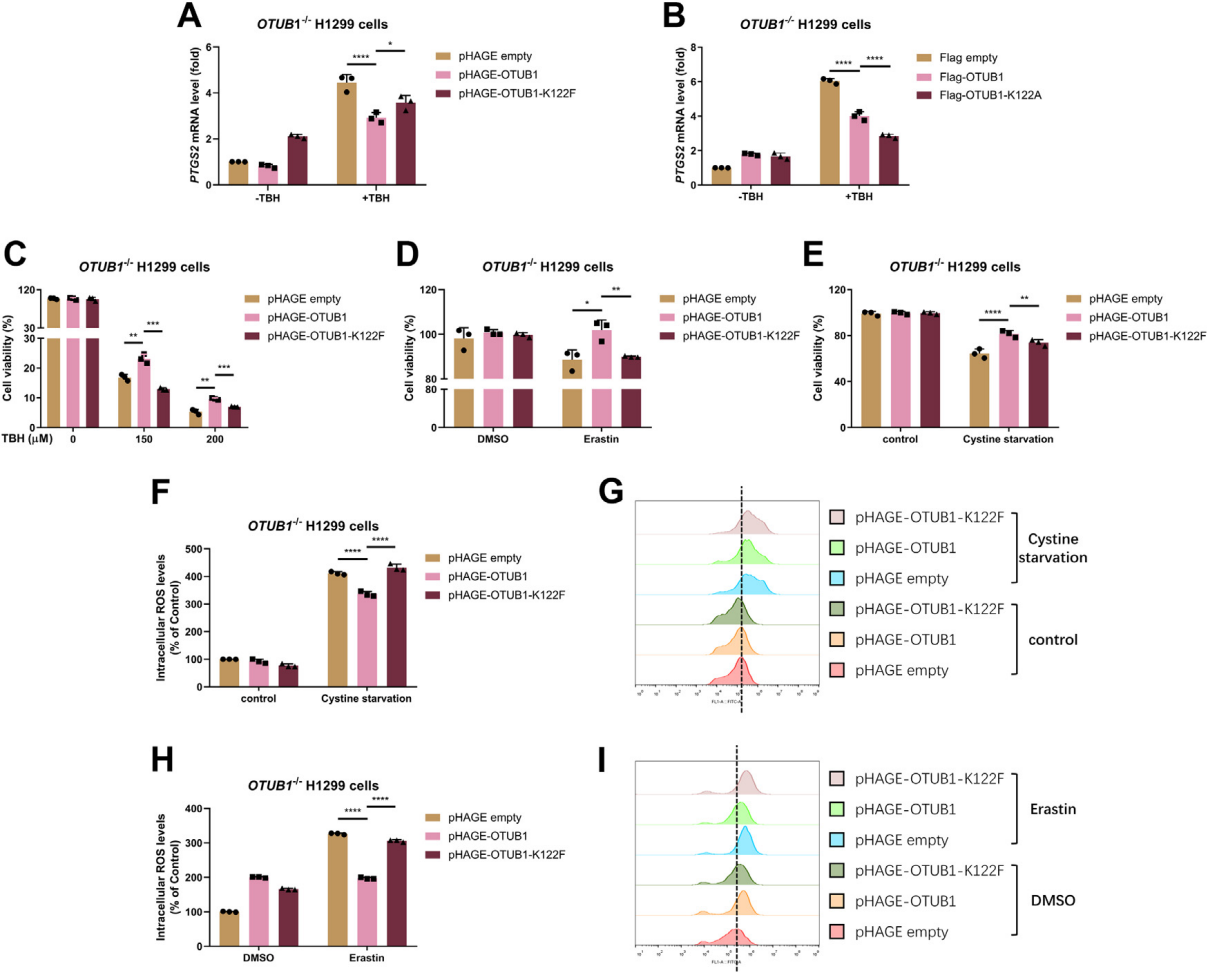
**SET7-mediated methylation of OTUB1 relieves its suppressive role on ferroptosis**. *A*, quantitative real-time PCR (qPCR) analysis of *PTGS2* mRNA in *OTUB1*-deficient H1299 cells (*OTUB1*^−/−^) reconstituted with *OTUB1* or its methylation-mimic mutant (OTUB1-K122F) by lentivirus and treated with or without TBH (100 μM) for 7 h. *B*, qPCR analysis of *PTGS2* mRNA in *OTUB1*-deficient H1299 cells (*OTUB1*^−/−^) reconstituted with *OTUB1* or its methylation-site mutant (OTUB1-K122A) by lentivirus and treated with or without TBH (100 μM) for 7 h. *C*, cell viability of *OTUB1*-deficient H1299 cells (*OTUB1*^*−/−*^) (n = 3) reconstituted with *OTUB1* or its methylation-mimic mutant (OTUB1-K122F) by lentivirus and treated with or without TBH (150 μM or 200 μM) for 7 h and examined by CCK8 assay. *D*, cell viability of *OTUB1*-deficient H1299 cells (*OTUB1*^*−/−*^) (n = 3) reconstituted with *OTUB1* or its methylation-mimic mutant (OTUB1-K122F) by lentivirus and treated with DMSO (as a control) or Erastin (20 μM) for 24 h and examined by CCK8 assay. *E*, cell viability of *OTUB1*-deficient H1299 cells (*OTUB1*^*−/−*^) (n = 3) reconstituted with *OTUB1* or its methylation-mimic mutant (OTUB1-K122F) by lentivirus and treated with or without cystine starvation for 24 h and examined by CCK8 assay. *F* and *G*, intracellular ROS in *OTUB1*-deficient H1299 cells (*OTUB1*^*−/−*^) (n = 3) reconstituted with *OTUB1* or its methylation-mimic mutant (OTUB1-K122F) by lentivirus and treated with or without Cystine starvation for 24 h and examined by flow cytometry. Quantification of the intracellular ROS levels in (*F*) and representative flow cytometry histogram in (*G*). *H* and *I*, intracellular ROS in *OTUB1*-deficient H1299 cells (*OTUB1*^*−/−*^) (n = 3) reconstituted with *OTUB1* or its methylation-mimic mutant (OTUB1-K122F) by lentivirus and treated with DMSO (as a control) or Erastin (20 μM) for 24 h and examined by flow cytometry. Quantification of the intracellular ROS levels in (*H*) and representative flow cytometry histogram in (*I*). Two-way ANOVA analysis; Data show mean ± SD; Tukey’s multiple comparisons test; ∗Adjusted *p* < 0.05, ∗∗Adjusted *p* < 0.01, ∗∗∗Adjusted *p* < 0.001, ∗∗∗∗Adjusted *p* < 0.0001; Data from three independent experiments. CCK8, Cell Counting Kit-8; ROS, reactive oxygen species; TBH, Tert-Butyl hydroperoxide.

Finally, we validated the role of SET7-mediated methylation of OTUB1 in ferroptosis. In response to TBH treatment, reconstitution of the WT OTUB1 in *OTUB1*^−/−^ H1299 cells suppressed the induction of *PTGS2* mRNA dramatically (Fig. 8*A*), but reconstitution of the methylation-mimic mutant of OTUB1 (K122F) relieved this suppressive effect (Fig. 8*A*). However, reconstitution of the methylation-site mutant of OTUB1 (K122A) strengthened this suppressive effect (Fig. 8*B*). As shown in Figure 8*C*, treatment with TBH caused dramatic cell death in a dose-dependent manner. Reconstitution of the WT OTUB1 reduced cells from TBH-induced ferroptosis, but reconstitution of the methylation-mimic mutant of OTUB1 (K122F) lost protective effect on TBH-induced ferroptosis (Fig. 8*C*). Similar results were obtained when the cells were treated with Erastin (Fig. 8*D*) or treated by Cystine starvation (Fig. 8*E*). In agreement with these observations, the ROS levels induced by Cysteine starvation or Erastin treatment were reduced significantly in *OTUB1*^−/−^ H1299 cells reconstituted with the WT OTUB1 but not in *OTUB1*^−/−^ H1299 cells reconstituted with the methylation-mimic mutant of OTUB1 (K122F) (Fig. 8, *F*–*I*).

Collectively, these data suggest that SET7-mediated methylation of OTUB1 relieves its suppressive role on ferroptosis induced by various stress.

## Discussion

Since the noncanonical activity of OTUB1 was reported (17), multiple studies have elucidated that OTUB1 noncanonically inhibits the ubiquitination of many proteins, indicating that OTUB1 regulates several biological processes in this manner (4, 6, 22, 32, 48, 49, 50). However, the regulation of this kind of activity of OTUB1 is barely understood (32, 34). Here, we identified that SET7-catalyzed lysine methylation of OTUB1 at K122 resulted in specifically inhibiting the noncanonical activity, but not the enzymatic activity of OTUB1. Therefore, this study enriched our understanding about the molecular regulation of OTUB1’s noncanonical activity.

The previous crystallographic analyses show that the residues amino-terminal to the OUT domain of OTUB1 (85–98 aa) are required for binding to UBC13∼Ub and inhibition of K63Ub synthesis (17), free ubiquitin binding to OTUB1's C-terminus allosterically triggers the formation of the E2/ubiquitin–binding alpha helix in OTUB1's N-terminus and that is essential for E2 inhibition (4), and deletion of the first 45 residues of OTUB1 abolishes the ability of OTUB1 to noncanonically inhibit E2s (4). It appears that the N-terminus and the OUT domain are critical for OTUB1 binding to and inhibition of UBC13. Here, we found that K122 of OTUB1 methylated by SET7 could affect OTUB1 binding to UBC13 obviously. However, K122 is neither located in the N-terminus nor located in the OUT domain of OTUB1. So, it is still enigmatic why K122 of OTUB1 methylated by SET7 could impair OTUB1 binding to UBC13. Further investigation of this question will open a new window for understanding the mechanisms of the regulation of OTUB1’s noncanonical activity.

It is noteworthy that many ubiquitin ligases as well as deubiquitinases are involved in various pathogenesis, such as cancer progression, autoimmune diseases, and inflammation (51). Screening of the efficient inhibitors or activators of ubiquitin ligases or deubiquitinases represent an active area for developing therapeutic drugs in the treatment of these diseases. If the enzymes act their roles without presenting catalytic activity, it will be relatively difficult to make therapeutic targeting of the enzymes. Notably, OTUB1 seems to be involved in some cancer progression or other pathogenesis by acting as a nonenzyme (1, 27). Thus, understanding the molecular regulation of noncanonical activity of OTUB1 will help to develop druggable targets for treating the related diseases. In this study, the finding of SET7-mediated methylation inhibiting the noncanonical activity of OTUB1 implicates that the inhibitor of SET7 might be utilized to enhance OTUB1 function for therapeutic purpose in the future.

## Experimental procedures

### Cell line and culture conditions

HEK293T and H1299 cells originally obtained from American Type Culture Collection were cultured in Dulbecco’s modified Eagle medium (VivaCell Biosciences) with 10% fetal bovine serum (FBS). *Set7*-deficient or WT MEF cells (*Set7*^*−/−*^ or *Set7*^*+/+*^) were established as described previously (46) and cultured in Dulbecco’s modified Eagle medium supplemented with sodium pyruvate (110 mg/l), 10% FBS, 1 × nonessential amino acids (Sigma), and 1% penicillin–streptomycin. The cell culture flasks, dishes, or plates were purchased from SORFA Life Science, and the cells were grown at 37 °C in a humidified incubator containing 5% CO_2_.

### Plasmid construction

The ORF of human SET7 was amplified from complementary DNAs (cDNAs) and subcloned into pCMV-HA (Clontech), pCMV-Myc (Clontech), pCMV-Flag (modified from pCMV-HA), pET32α (Novagen), and pHAGE-puromycin (obtained from Bo Zhong Lab) vectors. The enzymatically deficient mutant of SET7 (H297A) was subcloned into pCMV-Myc and pHAGE-puromycin vectors. The ORF of human OTUB1 was amplified from cDNAs and subcloned into pCMV-Flag, pCMV-HA, pCMV-Myc, pGEX-2T (GE Life Sciences), and pHAGE-puromycin vectors. The methylation-site mutant of OTUB1 (K122A) was subcloned into pCMV-Flag and pCMV-HA vectors. The methylation-mimic mutant of OTUB1 (K122F) was subcloned into pCMV-Flag, pCMV-HA, and pHAGE-puromycin vectors. The methylation-site mutant of OTUB1 (K122R) was subcloned into pCMV-HA and pGEX-2T vectors. The phosphorylation-site mutants of OTUB1 (S16E and S16A) was subcloned into pCMV-HA vector. The domain mutants of OTUB1 (OTUB1-N: 1–80 aa; OTUB1-C: 81–271 aa) were subcloned into pCMV-Flag vector. The ORF of human UBC13 was amplified from cDNAs and subcloned into pCMV-HA and pHAGE-puromycin vectors. The ORF of human TRAF3 was amplified from cDNAs and subcloned into pCMV-Myc vectors. All of these constructs were confirmed for accuracy by DNA sequencing.

### Antibodies and chemical reagents

Antibodies including anti-OTUB1 (#3783), anti-SET7 (#2825), anti-His (#9991), anti-Histone H3 (#4499), anti-UBC13 (#6999), anti-HA (#3724), anti-Ubiquitin (#3936), and normal rabbit IgG (#2729) were purchased from Cell Signaling Technology. Anti-OTUB1 (#ab270959) and anti-SET7 (#ab124708) antibodies were purchased from Abcam. Anti-β-actin (#AC026) antibody was purchased from ABclonal. Anti-Flag (#F1804) antibody was purchased from Sigma. Anti-HA (#901515) antibody was purchased from Covance. Anti-Myc (#SC-40) antibody was purchased from Santa Cruz Biotechnology. Anti-α-tubulin (#62204) and Alexa Fluor 488 goat anti-rabbit IgG (#A11008) were purchased from Thermo Fisher Scientific. Erastin (#HY-15763) was purchased from MCE. TBH solution (#B802372) was purchased from Macklin. CHX (#HY-12320) was purchased from MCE.

### Generation of anti-OTUB1-K122-me antibody

OTUB1-K122 site-specific methylation antibody (anti-OTUB1-K122-me antibody) was generated by using a human OTUB1 methylated peptide [FKAVSAKSK(me)EDLVSQ] as an antigen (ABclonal). After purifying the antibody with excess unmodified peptide (FKAVSAKSKEDLVSQ), the specificity of anti-OTUB1-K122-me antibody was verified by Dot blot and Western blot analysis.

### Immunoprecipitation and western blot

Coimmunoprecipitation and Western blot analysis were performed as described previously (44). Anti-Flag antibody–conjugated agarose beads (#A2220), anti-HA antibody–conjugated agarose beads (#A2095), and anti-Myc antibody–conjugated agarose beads (#A7470) were purchased from Sigma. Protein G Sepharose (#17-0618-01) was purchased from GE Health Care Company. The Fuji Film LAS4000 mini-luminescent image analyzer was used to photograph the blots.

### Identification of OTUB1 methylation site(s) by mass spectrometry

HEK293T cells were cotransfected with Flag-OTUB1 and Myc-SET7 plasmids. Cell lysate was immunoprecipitated with anti-Flag antibody–conjugated agarose beads overnight. Immunoprecipitated OTUB1 proteins were subjected to 8% SDS-PAGE gel, and OTUB1 bands were excised from the gel and analyzed by mass spectrometry in Protein Gene Biotech. The bands were digested as previously described (52).

The digested peptides were dissolved in 0.1% formic acid, separated on an online nano-flow EASY-nLC 1200 system with a 75 μm × 15 cm analytical column (C18, 3 μm, Thermo Fisher Scientific), and then analyzed on a Q Exactive HF-X mass spectrometer (Thermo Fisher Scientific). Peptides were eluted with the gradient of solvent B (0.1% formic acid in 80% acetonitrile) increased from 4% to 6% over 1 min, 6% to 10% in 2 min, 10% to 25% in 40 min, 25% to 45% in 10 min, climbed to 100% in 0.5 min, and then held at 100% for 6.5 min, all at a constant flow rate of 300 nl/min. The mass spectrometer was operated in data-dependent acquisition mode with full scans (m/z range of 350–1800) at 60,000 mass resolution using an automatic gain control target value of 3e6. The top 20 most intense precursor ions were selected for following MS/MS fragmentation by higher-energy collision dissociation with normalized collision energy of 28% and analyzed with 15,000 resolution in the Orbitrap. The dynamic exclusion was set to 25 s and the isolation width of precursor ion was set to 1.6 m/z. The maximum injection times were 20 ms and 50 ms for both MS and MS/MS, respectively. The intensity threshold was set to 5000.

The pFind software (version 3.1) (http://pfind.org/software/pFind/index.html) was employed for all MS/MS spectra analysis against the human protein database (https://www.ncbi.nlm.nih.gov/protein) combined with the reverse decoy database and common contaminants (53). Two missed cleavages were allowed for trypsin, and open-search algorithm in pFind was used. Methylation (K) was also set as variable modifications. The precursor and fragment ion mass tolerances were 20 ppm and 20 ppm, respectively. Minimum peptide length was set at 6, while the estimated false discovery rate threshold for peptide and protein were specified at maximum 1%.

### CRISPR-Cas9 KO cell lines

To generate the indicated gene knocked-out H1299 cells, sgRNA sequence were ligated into Lenti-CRISPRv2 plasmid and then cotransfected with viral packaging plasmids (psPAX2 and pMD2G) into H1299 cells. Six hours after transfection, medium was changed, and viral supernatant was collected and filtered through 0.45-μm strainer. Targeted cells were infected by viral supernatant and selected by 1 μg/ml puromycin for 2 weeks. The sgRNA sequence targeting *SET7* is TAGCGACGACGAGATGGTGG. The sgRNA sequence targeting *OTUB1* is GGTCCTGCTGAGCCATGA.

### Quantitative real-time PCR assay

Total RNAs were extracted using RNAiso Plus (TaKaRa Bio.) following the protocol provided by the manufacturer. cDNAs were synthesized using the Revert Aid First Strand cDNA Synthesis Kit (Thermo Fisher Scientific). MonAmp SYBR Green qPCR Mix (high Rox) (Monad Bio.) was used for quantitative real-time PCR assays. The primers for quantitative real-time PCR assays are described previously (46).

### Lentivirus-mediated gene transfer

HEK293T cells were transfected with pHAGE empty, pHAGE-OTUB1, pHAGE-OTUB1-K122F, pHAGE-UBC13, pHAGE-SET7, or pHAGE-SET7-H297A, together with the packaging vectors psPAX2 and pMD2G. Eight hours later, the medium was changed with fresh medium containing 10% FBS, 1% streptomycin–penicillin, and 10 μM β-mercaptoethanol. Forty hours later, supernatants were harvested and filtered through 0.45-μm strainer and then used to infect indicated cells.

### Ubiquitination assay

Ubiquitination assays were performed according to the protocol described previously with some modifications (46). Briefly, HEK293T cells were cotransfected with indicated plasmids for 24 h and then lysed by denatured buffer (6 M guanidine–HCl, 0.1 M Na_2_HPO_4_/NaH_2_PO_4_, 10 mM imidazole), followed by nickel bead purification and immunoblotting with the indicated antibodies.

### Immunofluorescence confocal microscopy

Immunofluorescence staining was conducted as previously described (54). Cells were seeded on glass coverslips and transfected with indicated plasmids. Then, the cells were fixed in 4% paraformaldehyde in PBS for 30 min at 25 °C. After washing three times by PBS, the slides were blocked in the blocking buffer (5% goat serum, 2 mg/ml BSA, 0.1% Triton X-100 in PBS) for 1 h and incubated with primary antibodies overnight at 4 °C, followed by incubation with Alexa Fluor 488 goat anti-rabbit IgG for 1 h at 25 °C. Subsequently, the slides were mounted with VECTASHIELD mounting medium containing DAPI and photographed with Leica SP8 laser scanning confocal fluorescence microscope.

### Nucleus and cytoplasm separation

Nucleus and cytoplasm separation was conducted with the Nuclear and Cytoplasmic Extraction Kit (#78833, Thermo Fisher Scientific) according to the protocol provided by the manufacturer. Then, the extracts were analyzed by Western blot analysis. To ensure the efficiency of fraction separation, anti-α-tubulin antibody was employed to monitor cytoplasmic proteins and anti-Histone H3 antibody was used to monitor nuclear proteins.

### Enzymatic activity assay

HEK293T cells were transfected with Flag-OTUB1 or Flag-OTUB1-K122F plasmid. Twenty four hours later, total cell lysates were immunoprecipitated with anti-Flag antibody–conjugated agarose beads overnight and then eluted with 3 × Flag peptides. For enzymatic activity, 2 μg K48 di-Ub chain (#SI4802, LifeSensors) was added to the reaction buffer containing eluted Flag-OTUB1 or Flag-OTUB1-K122F and then incubated at room temperature for indicated time. The reaction was terminated by the addition of 2 × SDS loading sample buffer. The samples were separated by SDS-PAGE, and the ubiquitin cleavage activity of OTUB1 was detected by Western blot analysis using anti-ubiquitin antibody.

### Cell viability assay

Cells were seeded into 96-well plates and cultured as indicated. After adhesion, cells were treated with the different ferroptosis inducers. Then, cell viability was assessed with the Cell Counting Kit-8 assay according to the manufacturer’s instructions. The absorbance was measured using the ELx800 microplate reader (BioTek Instruments) at 450 nm and relative cell viability was calculated.

### Intracellular ROS assay

Intracellular ROS was detected using CM-H2DCFDA (General Oxidative Stress Indicator) (#C6827, Thermo Fisher Scientific). After treatment as indicated, the cells were harvested and incubated with 1 μM CM-H2DCFDA for 1 h at 37 °C. Then, the cells were washed three times with ice-cold PBS and intracellular ROS were detected by flow cytometry analysis.

### Statistical analysis

GraphPad Prism 8.0 software (GraphPad Software) was used for all statistical analysis. Differences between experimental and control groups were determined by unpaired two-tailed Student’s *t* test (where two groups of data were compared) or two-way ANOVA analysis (where more than two groups of data were compared). *p* values or adjusted *p* values less than 0.05 were considered statistically significant.

## Data availability

Raw mass spectrometry data have been deposited to the ProteomeXchange Consortium (http://proteomecentral.proteomexchange.org) *via* iProX partner repository with the dataset identifier IPX0005780001 (55). The location is https://www.iprox.cn//page/subproject.html?id=IPX0005780001. All peptide sequences assigned are listed in Supplementary Table S1. Further information and requests for resources and reagents should be directed to and will be fulfilled by Xing Liu and Wuhan Xiao.

## Supporting information

This article contains supporting information.

## Conflict of interest

The authors declare that they have no conflicts of interest with the contents of this article.

## Figures and Tables

**Original Western Blot Results-revise:**